# Implications and Considerations of Dental Materials in MRI: A Case Report and Literature Review

**DOI:** 10.1155/2020/8891302

**Published:** 2020-12-15

**Authors:** Brenton J. Wilson, Phoebe E. O'hare, John Zacariah

**Affiliations:** ^1^James Cook University, Cairns, Australia; ^2^Oral Health Services Northern Territory, Australia

## Abstract

Magnetic resonance imaging (MRI) has an increasing role as a diagnostic imaging modality. Dental materials have important implications on the use of MRI as a diagnostic imaging modality. A case of a dislodged crown while in an MRI machine prompted a review of the literature for the implications and considerations of dental materials with magnetic resonance technology. An understanding of the basic physics involved in magnetic resonance is required to appreciate the relevance of dental materials in an MRI scanner. This case report supported by a literature review recommends assessing a patient's crown retention prior to and after MRI scanning.

## 1. Introduction

Magnetic resonance imaging (MRI) has an increasing role as a diagnostic imaging modality in Australia. In 2015, there were 41 MRI scans performed per 1000 population [1]. Although MRI technology currently has limited uses in dentistry, such as evaluation of temporomandibular joint anatomy, dental patients are increasingly exposed to MRI for medical indications. There are a number of reasons why MRI technology is becoming more frequently utilised internationally, including increased accessibility, the absence of ionizing radiation, and superior soft tissue resolution compared to other imaging modalities [2].

Dental materials have important implications on the use of MRI as a diagnostic imaging modality. A case of a dislodged crown while in an MRI machine prompted a review of the literature for the implications and considerations of dental materials with magnetic resonance technology, with particular relevance to the Australian setting. These implications can be divided into radiofrequency-driven heating, magnetically induced displacement forces, and image artefact [3]. An understanding of the basic physics involved in magnetic resonance is required to appreciate the relevance of dental materials in an MRI scanner.

## 2. Case Study

A 74-year-old lady presented to a public dental clinic in the Northern Territory with a dislodged crown from tooth 11. She was undergoing an MRI as part of her workup for a brain tumour. As the machine started up, she felt the crown “pull” on her tooth and dislodge into her mouth. There was no documentation of the metallic composition of this porcelain fused metal crown, which was constructed and cemented in Indonesia approximately 8 years ago. She has had no issues with the crown until this MRI. She presented to the dental clinic within 4 hours of dislodgement.

On examination, the crown preparation was in good condition, except for a small area of marginal caries on the distal. The patient opted to have the caries excavated and restored with a glass ionomer cement (GIC) and the crown recemented with a resin-reinforced luting GIC. Given the need for ongoing MRI scans as part of her brain tumour monitoring and consequent risk of recurrent dislodgement, it was recommended that she have an alternative restoration considered in the future. Signed informed consent was sought from this patient to present her case.

## 3. Discussion

The temporal relationship between the crown dislodging and commencement of the MRI mechanism is indicative as the probable cause of the dislodgement. Other causes of fixed prosthodontic retention loss include adhesive or cohesive failure of cement, excessive crown taper, short clinical crown length, or recurrent caries [4]. These factors may contribute but would be significantly coincidental given the timing of retention failure with the diagnostic scan. In our review of literature, we identified only one other similar case in a published letter to the editor, where a patient felt the MRI “nearly pulled his crown out” [5]. In this case, the cast core and crown were found to be made with 82% nickel content. Potential reasons for the scarcity of published literature are the relatively recent introduction of MRI to Australia in 1998 in addition to the change in profile of dental materials globally in recent times.

MRI technology relies on a strong uniform magnetic field that results in uniform alignment of protons within its field. A magnetic vector along the axis of the MRI scanner is consequently generated, and radiofrequency waves are then directed towards these protons. The returning waveforms are detected by radiofrequency receptors and converted into images reflecting the different tissue compositions [2]. The strength of the magnetic field can have a “projectile effect,” by pulling ferromagnetic materials. Due to this strong magnetic field, stringent safety mechanisms are practised in Australia to prevent harm and injury. The Royal Australian and New Zealand College of Radiologists MRI safety guidelines require patients to be screened for potentially hazardous items before entering the magnetic field zone. Metal objects are removed by the patient prior, and any medical implantable devices need to be checked for their compatibility with magnetic resonance [6]. The three commonest ferromagnetic metals are iron, nickel, and cobalt.

### 3.1. Dental Materials

There is a wide array of materials used in dentistry, many left in the mouth long-term, such as implants and dental crowns. Ferromagnetic metals are commonplace in orthodontic brackets and wires, dentures, and crowns. The most common issue with dental materials is their susceptibility to creating artefacts and distortions in MRI. These have been divided by Tymofiyeva et al. into compatible, compatible I, and noncompatible based on the resultant distortion of the image (Table 1) [7].

With regard to displacement of the crown in this case, there are two potential methods of displacement. The first is that radiofrequency energy is absorbed, resulting in heating, compromising the adhesion of the crown to the tooth structure. Hasegawa et al. investigated the temperature increase of fixed prosthodontic appliances in MRI scanners and found an increase of <2°C, which would have negligible effect on the luting cement.

The second factor is the magnetically induced displacing forces created by the MRI machine. Ferromagnetic components of the patient's crown may have created enough displacing forces to overcome the already compromised retentive force of the luting cement on the crown [8]. For this reason, Chockattu et al. recommend that ferromagnetic containing dental prostheses are checked for retention prior to and after the MRI scan [3].

### 3.2. The Australian Context

Of potential relevance in this case is the Indonesian origin of the patient's crown. The material used in the crown is unclear, and the patient did not allow permission for this to be removed for testing. Medical and dental tourism is an increasing trend around the world, whereby patients seek lower-cost medical and dental care overseas in countries such as Indonesia [9]. In many countries, the dental industry, including the use of dental materials, is subject to approval by the Therapeutic Goods Administration (TGA). Dental materials require testing certificates for approval by TGA and consequently do not require an implantable card and considered safe for MRI. Other implantable materials such as cardiac defibrillators and cochlear implants that do not meet MR requirements necessitate the patient to be given a card highlighting that the material is MR conditional or MR unsafe, depending on the field condition of the MRI [10].

### 3.3. Conclusions/Recommendations

With the increasing use of MRI technology, dentists should be aware that dental materials affect MR images and MR technology affects dental materials. The most significant impact is the effect on image quality. Recommendations from the literature are based predominately on scientific ex vivo studies on the effect of MR on materials as well as small retrospective studies on image quality. With regard to improving image quality, removable dentures and orthodontic wires should be removed where possible prior to scanning, particularly when the head and neck region is being imaged. This case report highlights a case where a crown has dislodged at the commencement of MRI scanning and the potential mechanisms of this. In terms of displacement and heating, the literature is limited to ex vivo studies of dental materials and appliances placed in MRI scanners. These studies have found limited risk of dental materials causing damaging heating or significant displacement in the magnetic field. This case report, however, supports the Chockattu et al. recommendation of assessing a patient's crown retention prior to and after MRI scanning.

## Figures and Tables

**Table 1:** Dental materials and impact on MR image artefact.

Classification	Clinical significance	Materials
Compatible	No image artefact or distortion	Glass ionomers, resin, gutta percha, zirconium dioxide, some composites
Compatible I	Limited image artefact or distortion localised to imaging site	Amalgam, gold alloy, gold ceramic crowns, titanium, some composites
Noncompatible	Significant image distortions, even when imaging site distant from material	Stainless steel, cobalt-chrome

## Data Availability

Data may be accessed on request from the corresponding author, Dr. Brenton Wilson.
